# The Effect of High Dose Rate Interstitial Implant on Early and Locally Advanced Oral Cavity Cancers: Update and Long-Term Follow-Up Study

**DOI:** 10.7759/cureus.7910

**Published:** 2020-05-01

**Authors:** Parthasarathy Vedasoundaram, Abhishek Raghava KS, Kannan Periasamy, Gangothri Selvarajan, Sudhakar K, Saravanan Kandasamy, Seenisamy R, Aravind Kumar

**Affiliations:** 1 Radiation Oncology, Jawaharlal Institute of Postgraduate Medical Education and Research (JIPMER), Puducherry, IND; 2 Oncology, Jawaharlal Institute of Postgraduate Medical Education and Research (JIPMER), Puducherry, IND; 3 Radiation Oncology, Postgraduate Institute of Medical Education and Research (PGIMER), Chandigarh, IND; 4 Medical Oncology, Adyar Cancer Institute, Chennai, IND; 5 Medical Physics, Jawaharlal Institute of Postgraduate Medical Education and Research (JIPMER), Puducherry, IND; 6 Radiation Oncology, Puducherry Cancer Trust Hospital, Puducherry, IND

**Keywords:** oral cavity cancer, squamous cell carcinoma, external beam radiation therapy, high dose rate interstitial brachytherapy, organ preservation

## Abstract

Introduction

Brachytherapy, with or without external beam radiation therapy (EBRT), can be an alternative to surgery for organ preservation in early and locally advanced oral cavity cancers. This study aims to evaluate the effect of high dose rate (HDR) interstitial brachytherapy on early and locally advanced squamous cell carcinoma (SCC) of the oral cavity when used alone or as a boost to EBRT.

Methods

A total of 125 patients with histologically proven stage T1-3/N0-1 SCC of the oral cavity were included in the study. A total of 15 patients with stage I disease received an interstitial implant dose of 3,850 cGy at 350 cGy per fraction, two fractions a day. Another 53 patients had stage II, and 57 patients had stage III disease; these patients received EBRT of 50 Gy in 25 fractions along with an HDR brachytherapy boost of 21 Gy in seven fractions of 3 Gy per fraction twice daily. The stage III patients also received concurrent chemotherapy with injections of cisplatin (70 mg/m^2^) given every three weeks for three days in divided doses. All node-positive patients received a boost to the node of up to 64 Gy by external beam radiation. Disease response rates, five-year disease-free survival rates, and toxicities were analyzed.

Results

The median follow-up was 60 months. Among the patients, 103 (82.4%) had a complete response, while 22 (17.6%) had residual disease and were referred for surgical salvage. The five-year disease-free survival was 100% in stage I, 83% in stage II, and 77.2% in stage III; 4% of patients developed grade 3 acute skin toxicity and 23.2% developed acute grade 3 mucositis. Eleven patients died during the follow-up period. Two patients died due to myocardial infarction but had achieved a complete tumor response. One patient had pulmonary tuberculosis and died due to fulminant infection after three years of disease-free survival period. One patient developed a second primary in the brain stem that presented with quadriplegia and expired. Seven patients died due to the progression of the initial disease.

Conclusions

Proper brachytherapy technique and meticulous planning can minimize the toxicity while providing better tumor control and achieve high local control rates. Brachytherapy, with or without EBRT, can be a surrogate to surgery in early oral cavity cancers as it can achieve organ preservation while providing good functional outcomes.

## Introduction

Oral cavity cancer is the 16th most common cancer worldwide and the fourth most common cancer in low-to-middle-income countries. According to the Global Cancer Observatory 2019 cancer statistics, 0.35 million new oral cavity cancers and 0.17 million oral cavity cancer-related deaths were reported in 2018. In 2019, the global age-standardized incidence and mortality rates for oral cavity cancers were four and two per 100,000 populations, respectively [1].

Though surgery with or without adjuvant radiation has been the preferred approach to treat oral cavity cancers, the National Comprehensive Cancer Network (NCCN) 2019 guidelines state that radiation with or without chemotherapy can provide a better alternative [2-4]. The role of radiation has been well established for oral cavity cancers. The challenge in planning radiotherapy is to achieve a high dose to the target without an increase in toxicities for healthy tissue. Brachytherapy has shown good efficacy in early cancers of the head and neck [5]. Studies by Patra et al. and Rudoltz et al. have shown better local control with a high dose rate (HDR) brachytherapy boost compared to external beam radiation therapy (EBRT) alone [6,7]. This was true even in oral cavity cancers of stages T3 and T4. Our study was performed to assess the benefit of brachytherapy, both as the sole modality of treatment in early oral cavity cancers and with EBRT as a boost in locally advanced oral cavity cancers.

## Materials and methods

For our study, we considered 134 patients with histologically proven squamous cell carcinoma (SCC) of the oral cavity who attended the outpatient department of Radiation Oncology at the Regional Cancer Centre in Jawaharlal Institute of Postgraduate Medical Education and Research (JIPMER), Puducherry, India, from November 2008 to February 2015. Of these, nine patients did not appear for follow-up and were therefore excluded from the study. The results of 125 patients were analyzed. Among these, 77 were men, and 48 were women. Ages ranged from 32 to 73 years (mean: 53.91 years). Patients with stages T1-T3/N0-N1 cancers and an Eastern Cooperative Oncology Group (ECOG) performance score of up to 2 were recruited. A complete staging workup including a thorough physical examination, a biopsy of the primary site, fine needle aspiration cytology of clinically identified lymph nodes, chest X-rays, contrast-enhanced CT scans of head and neck (if needed), and baseline blood counts and biochemistry were done for all the patients. All patients underwent a pre-anesthetic check-up, and their fitness for anesthesia was obtained.

Primary HDR brachytherapy with a dose of 38.5 Gy at 3.5 Gy per fractions for 11 fractions, twice daily at six hours apart was considered in 15 patients with stage I disease. A total of 53 patients with stage II received EBRT and brachytherapy boost alone, while 57 patients with stage III disease also received concurrent chemotherapy with cisplatin injection at 70 mg/m^2^ in three divided doses, three weeks apart, along with EBRT. EBRT was delivered at a dose of 50 Gy in 25 fractions of two Gy per fraction, five fractions per week for five weeks. An additional HDR brachytherapy boost of 21 Gy, given in seven fractions of 3 Gy each, twice daily at six hours apart, was given within two to four weeks of EBRT.

Field set-up and simulation were done before EBRT for all stage II and stage III cases. An EBRT dose of 44 Gy without off-cord and 6 Gy by off-cord, for a total dose 50 Gy over 25 fractions in 2 Gy per fraction per day, was given to the primary site as well as the regional lymph nodes of the upper neck. A total of 50 Gy over 25 fractions was delivered to the low anterior neck as elective nodal irradiation for uninvolved neck nodes. This was followed by an HDR interstitial brachytherapy boost two to four weeks later. Treatment was delivered by a CLINAC IX/CLINAC 600C (Varian Medical Systems, Palo Alto, CA). All node-positive patients received a nodal boost of up to 64 Gy by external beam radiation, and these patients received brachytherapy after completion of the entire EBRT schedule.

Brachytherapy procedure

Under general anesthesia by nasal intubation, flexible catheters were placed as a double plane implant. On the second day after the procedure, a non-contrast planning CT scan of the implanted region with 1-mm thick slices was taken using a Somatom Spirit (Siemens AG, Munich, Germany) CT scan. The images were then transferred to either treatment planning systems from Oncentra MasterPlan (Elekta, Stockholm, Sweden) or BrachyVision (Varian Medical Systems, Palo Alto, CA). The clinical target volume (CTV) was delineated, and the applicators were reconstructed. At the tip of all the applicators, a reference point was inserted. Dose optimization was performed by adjusting dwell times in individual dwell positions to ensure that at least 90% of the CTV received the prescribed dose. For generating dose distributions, the American Association of Physicists in Medicine (AAPM) Task Group No. 43 (TG-43) protocol was used, and a cumulative dose-volume histogram was used for plan evaluation [8].

Follow-up

Patients were assessed for toxicity both during treatment and on follow-up based on Radiation Therapy Oncology Group (RTOG) criteria; toxicities were recorded and graded per these criteria. Complete clinical disappearance of the disease was considered as locally controlled. Any suspicious indurated or ulcerated lesions were subjected to biopsy. Biopsy-proven positive patients were considered as patients with residual disease and were sent for surgical salvage. All patients were followed up every two months in the first year, every three months for up to five years, and yearly after that. Additionally, patients were asked to report any specific or non-specific symptoms related to the cancer site or in the irradiated area.

Statistical analysis

The data related to the patient’s clinical characteristics, such as the local control and toxicity profiles, were presented as frequencies and percentages. A comparison of these variables between the groups was carried out using a chi-squared or Fischer’s exact test. All statistical analyses were carried out at a 5% level of significance; a p-value of <.05 was considered statistically significant.

Ethical considerations

Informed consent was obtained from all participants after a proper explanation of the treatment procedure in their language. Ethical clearances were obtained from the JIPMER Institute Ethics Committee per SEC/2011/4/101 and JIP/IEC/2014/1/255.

## Results

Patient and tumor characteristics

The patients and tumor characteristics are summarized in Table 1. It shows the summary of patient profiles including age, gender, habits, and tumor stage and growth pattern. Of the 125 eligible patients, 68 patients had early-stage oral cancer (I and II), and 57 patients had locally advanced (stage III) disease. Among these, 15 were T1, 62 were T2, and 48 were T3. Fourteen of 125 patients presented with a clinically palpable lymph node. Of the lesions, 60% was in the anterior two-thirds of the tongue, 34.4% was in the buccal mucosa, and the remaining 5.6% was on the floor of the mouth.

**Table 1:** Patient characteristics *Age group: 32-73 years (mean: 53.91) DM: diabetes mellitus; HTN: hypertension; MD: moderately differentiated; SCC: squamous cell carcinoma; WD: well-differentiated

Characteristics	Number	Percentage
Number of patients*	125	
Men	77	61.6
Women	48	38.4
Comorbidities		
DM	18	14.4
HTN	15	12
None	56	44.8
Personal history		
Alcohol	20	16
Tobacco chewing and smoking	59	47.2
Both alcohol and tobacco	15	12
None	46	36.8
Growth pattern		
Proliferative	75	60
Infiltrative	40	32
Both	10	8
Biopsy report		
SCC-WD	85	68
SCC-MD	40	32
Site		
Anterior two-thirds of the tongue	75	60
Buccal mucosa	43	34.4
Floor of the mouth	7	5.6
Stage		
I	15	12
II	53	42.4
III	57	45.6
T-Stage		
1	15	12
2	62	49.6
3	48	38.4
N-stage		
0	111	88.8
1	14	11.2

Treatment outcomes

**Figure 1:**
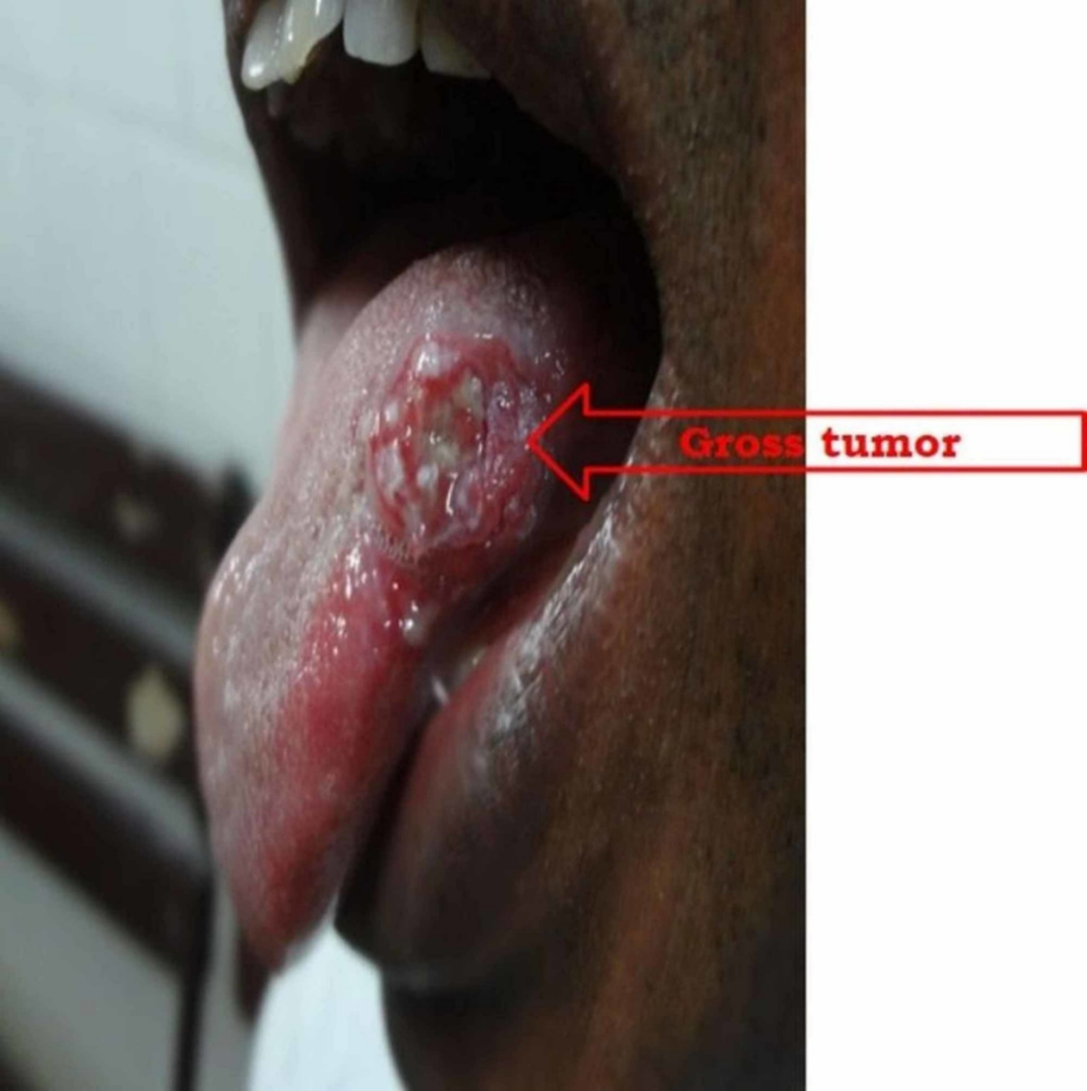
A pre-treatment gross tumor seen in carcinoma in the anterior two-thirds of the tongue

**Figure 2:**
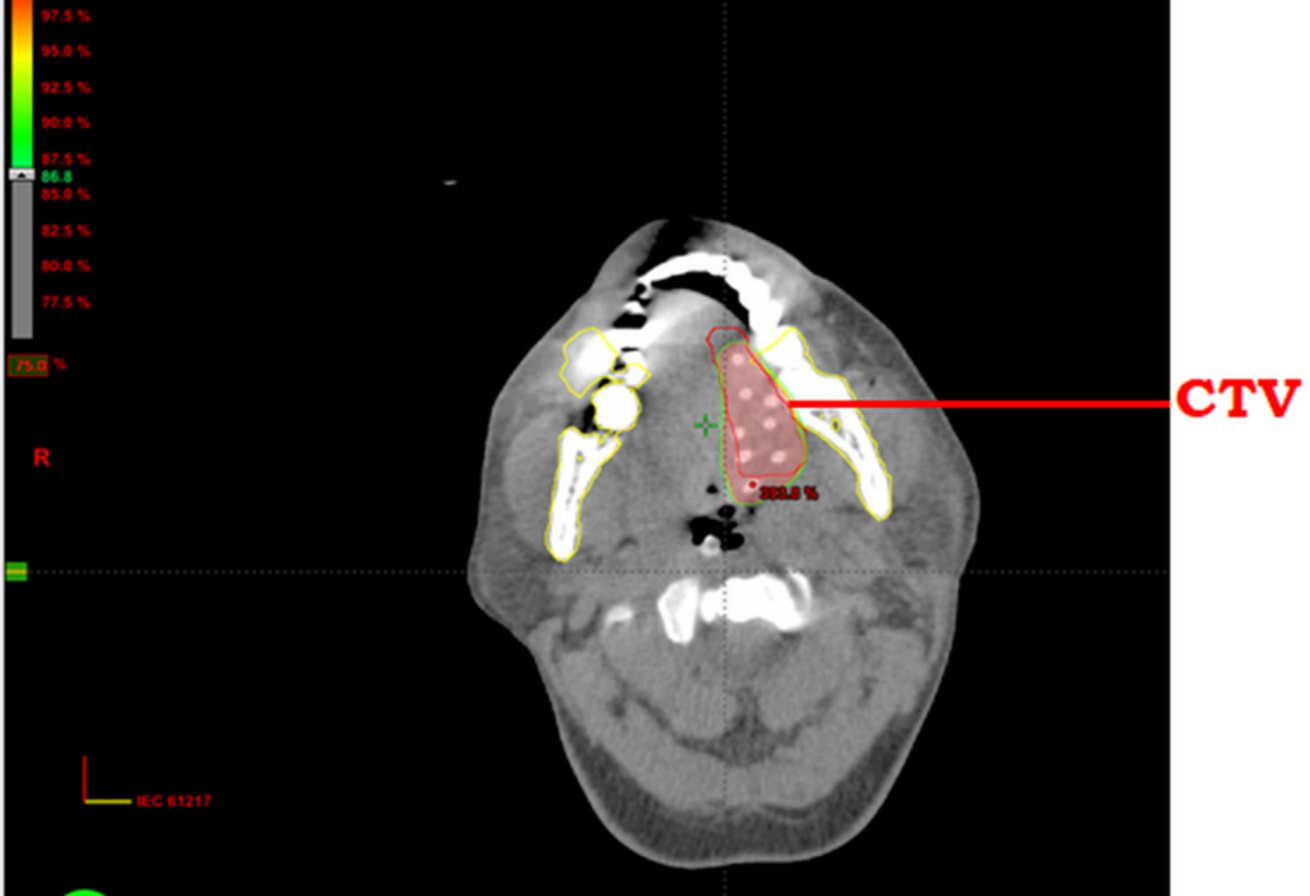
Clinical target volume and dose color wash in the left lateral side of the anterior two-thirds of the tongue showing adequate target volume coverage CTV: clinical target volume

**Figure 3:**
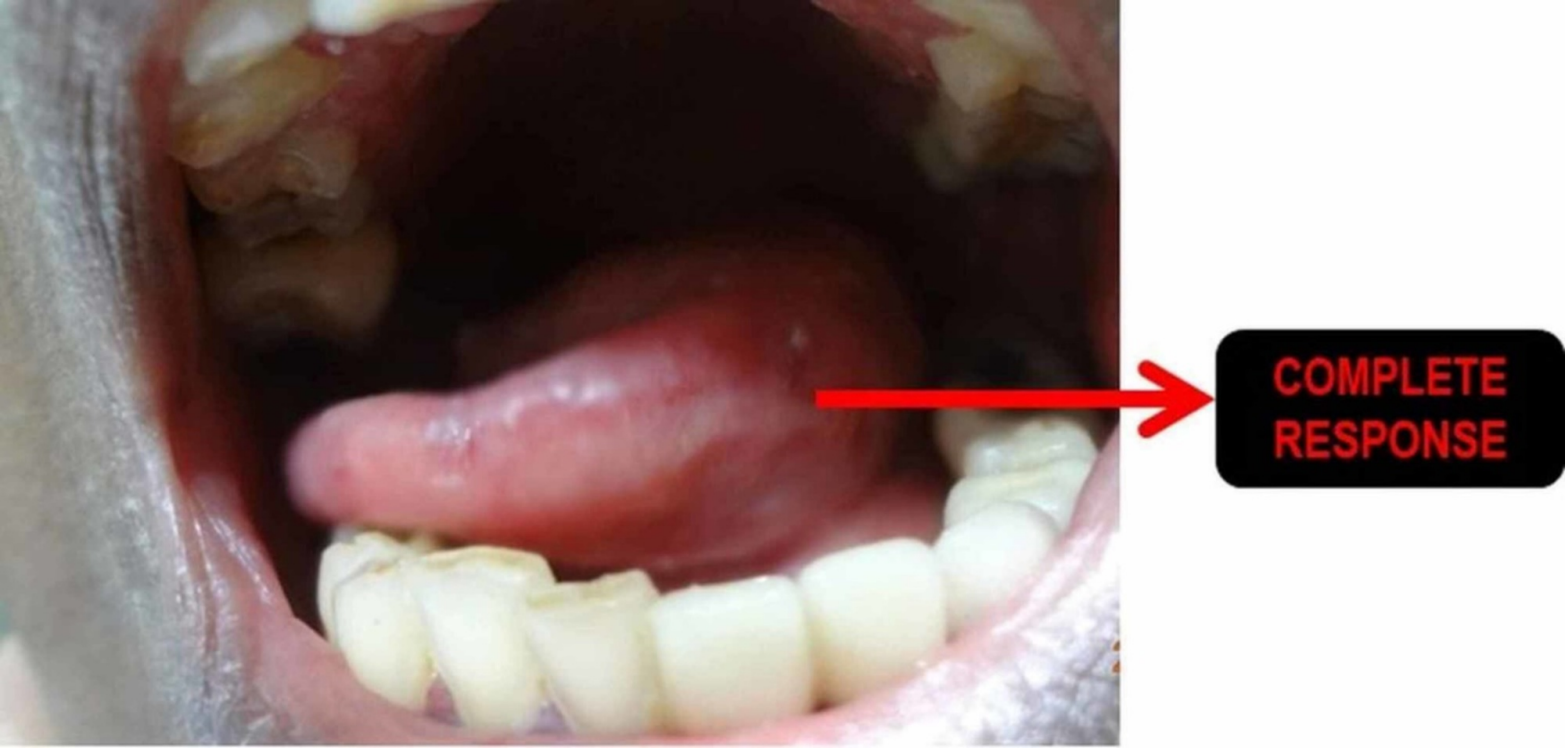
A complete response seen in the anterior two-thirds of the tongue of the same patient

The median follow-up was 60 months. The overall treatment outcomes are shown in Table 2. Among the participants, 103/125 had a complete clinical response (82.4%), and 22/125 (17.6%) were found to have a biopsy-proven residual tumor. Five-year disease-free overall survival (OS) was 100% for stage I, 83% for stage II, and 77.2% for stage III. Twenty-two patients had residual disease. In the complete response group, 57% were at the early stages (I and II), while 43% were at a locally advanced stage (III). Figure 1, Figure 2, and Figure 3 show the pre-treatment evaluation, CTV coverage, and post-treatment follow-up, respectively, of a patient with carcinoma of the tongue. Subgroup analysis for the nodal stage of the tumor found a significant effect on local control following treatment (p-value of 0.01), as shown in Table 3.

**Table 2:** Treatment outcomes in relation to cancer stage

Outcome	Stage I		Stage II		Stage III		P-value
	N	%	N	%	N		
Complete response	15	100	44	83	44	77.2	>0.05
Residual disease	0	0	9	17	13	22.8	
Total	15	100	53	100	57	100	
Pearson’s chi-squared test							0.14

**Table 3:** Treatment outcomes in relation to nodal stage

Outcome	N0		N1		Total	P-value
	N	%	N	%		
Complete response	95	85.5	8	57	103	<0.05
Residual disease	16	14.5	6	43	22	
Total	111	100	14	100	125	
Fisher's exact test						0.01

Residual disease

Those with residual diseases proven by biopsy on follow-up were referred to the surgical oncology department for salvage surgery. Seven patients did not undergo surgery as the disease progressed very fast; for them, palliative chemotherapy with cisplatin injections and paclitaxel was considered, but they succumbed due to the progression of the disease. Other patients did not appear for follow-up after surgical salvage, and their disease status is unknown.

Effects of tobacco and alcohol

Among the participants, 47.2% had a history of tobacco intake, 16% consumed alcohol, and 12% reported both. Subgroup analysis for tobacco intake found a significant effect on local control (p-value of 0.03). However, subgroup analysis for alcohol did not find any significant impact on local control.

Toxicities

Table 4 shows the various acute and late toxicities observed in these patients. Toxicities were graded using the RTOG criteria. Out of 125 patients, 4% developed grade 3 acute skin toxicity and 23.2% developed acute grade 3 mucositis; 11.2 % were found to have grade 2 subcutaneous fibrosis, and 40% developed both acute and chronic grade 2 xerostomia. Five patients developed bleeding at the time of implant catheter removal after brachytherapy treatment; this was effectively managed by compression for a few minutes and ice packs; no one required surgical intervention or blood transfusion. Soft tissue necrosis was seen in 5.6% of patients and 4.8% of patients developed osteoradionecrosis.

**Table 4:** Toxicities observed during radiation therapy and on follow-up *Grading based on the Radiation Therapy Oncology Group criteria

Toxicities		N (125)	%
Acute skin toxicity	G0*	15	12
	G1*	84	67.2
	G2*	21	16.8
	G3*	5	4
Acute mucositis	G0*	15	12
	G1*	36	28.8
	G2*	45	36
	G3*	29	23.2
Xerostomia	G0*	45	36
	G1*	30	24
	G2*	50	40
Acute vomiting	G0*	89	71.2
	G1*	29	23.2
	G2*	7	5.6
Acute nausea	No	72	57.6
	Yes	53	42.4
Subcutaneous fibrosis-chronic	G0*	40	32
	G1*	71	56.8
	G2*	14	11.2
Soft tissue necrosis		7	5.6

Mandible volume dosing

Mandible volume receiving doses of 200%, 150%, and 100% of the prescribed dose during brachytherapy is represented by V200%, V150%, and V100%, respectively. The mean values for these parameters are shown in Table 5.

**Table 5:** Mean values for V200%, V150%, and V100% of the mandible in the study group cc: cubic centimeters; SD: standard deviation; V200%: volume receiving 200% of the dose; V150%: volume receiving 150% of the dose; V100%: volume receiving 100% of the dose

Mandible volume		
Parameters	Mean	±SD
V200% (cc)	0.049457	0.098911
V150% (cc)	0.137235	0.234455
V100% (cc)	1.077939	1.035561

## Discussion

The primary aim of the treatment is to have a complete cure with minimal functional deformities and a good cosmetic outcome. Surgery often results in the removal of an organ completely or partially, depending upon the stage of cancer, leading to a poor cosmetic outcome. Radiotherapy has the advantage of preserving the normal anatomy of the organ with a good functional outcome and provides better cosmesis, making it an effective alternative to surgery. Goffinet et al. have demonstrated that EBRT plus an interstitial brachytherapy boost resulted in better two-year relapse-free survival than surgery plus adjuvant radiotherapy (76% vs. 36%, respectively) [9]. Sresty et al. have reported that interstitial brachytherapy treatment confers more dose homogeneity when compared with intensity-modulated radiation therapy and lesser dose to critical structures [10]. Additionally, planning time was much lower for most cases. They concluded that interstitial brachytherapy was an ideal option for high-dose delivery exclusively to the primary tumor volume while limiting adverse effects.

For stage I oral cavity cancers, brachytherapy alone can be considered based on the results from the present study. Housset et al. have reported that a brachytherapy boost is superior to external radiation alone for T1 and T2 tumors [11]. Guinot et al. have reported a local control rate of 94% in T1 and 84% in T2 when treated with a brachytherapy boost and a complete response in all patients who were treated with brachytherapy alone with corresponding morbidities of osteoradionecrosis (4%) and soft tissue necrosis (16%) [12]. In our study, complete response rates in stage I and stage II tumors were 100% and 83% respectively. The risk of soft tissue necrosis within the implant volume in patients receiving brachytherapy, as mentioned by Mazeron et al., was 10-30% [13]. In our study, soft tissue necrosis and osteoradionecrosis were seen in 5.6% and 4.8% of patients respectively.

The optimal management in stage III oral cavity malignancies are either surgical resection followed by adjuvant radiotherapy/chemoradiotherapy or radical chemoradiation. Foster et al. have reported 78.6% local control rates in stage III/IV oral cavity SCC with definitive chemoradiation [14]. These rates are almost comparable to 77.2% in the present study. Retrospective studies that compared the efficacy of definitive chemoradiation to postoperative radiotherapy have shown conflicting results. For instance, Spiotto et al. have reported a statistically significant three-year superior OS for patients with stage III disease receiving postoperative radiotherapy compared to definitive chemoradiation [15]. Tangthongkum et al. have found the five-year OS to be statistically equivalent for patients with stage III disease receiving definitive chemoradiation, compared to postoperative radiotherapy [16]. The differences in the outcomes from these studies can be attributed to patient-related factors as most of the patients who were medically inoperable or with unresectable disease received definitive chemoradiation.

CTV should not be over-ambitious. CTV should be close to the catheter in order to achieve ideal dose coverage. It is desirable to implant more flexible tubes equidistant from other than restricting the number of catheters. This gives more room for uniform dose delivery and better homogeneity index (DHI) and conformal index (COIN). According to the American Brachytherapy Society recommendations, a coverage index (CI) of 1, DHI of more than 0.75, and an external volume index (EVI) of 0 should be achieved with more than 90% of dose delivered to >90% target volume [17]. In this study, the mean coverage index was 0.7 (ideal = 1), DHI was 0.72 (ideal = 0.75), dose-nonuniformity ratio (DNR) was 0.2 (ideal = 0.1), COIN was 0.75 (ideal = 1), and the EVI was 0.06 (ideal = 0.05). All parameters were well within the recommended ranges (Table 6).

**Table 6:** Brachytherapy indices CI: coverage index; COIN: conformal index; CR: complete response; DHI: dose homogeneity index; DNR: dose nonuniformity ratio; EVI: external volume index; R: residual disease; SD: standard deviation

Indices	CR		R	
	Mean	SD	Mean	SD
CI	0.77110	0.07138	0.77260	0.4662
DHI	0.72351	0.12418	0.71605	0.16255
COIN	0.75746	0.09471	0.76863	0.05664
DNR	0.26616	0.12597	0.31469	0.15651
EVI	0.06775	0.06144	0.04588	0.01453

The treatment was well tolerated; the most common adverse events observed were dermatitis and mucositis. Among the patients, 67.2% had grade I dermatitis, and 28.8% had grade I mucositis. Fully 85% of the patients with tumors in anterior two-thirds of the tongue provided a history of a burning sensation over the dorsum of the tongue. They were treated with zinc-containing multivitamin tablets and antiseptic oral rinses; 4% of all patients developed grade III skin toxicity during EBRT. For these patients, radiation had to be briefly withheld to allow healing. Fully 92% of the tongue cancer patients provided a history of a loss of taste sensation that returned to normal during the six month-to-one-year follow-up period. Soft tissue necrosis was seen in seven patients. All patients were kept on a follow-up schedule for six months. The necrosis resolved in five patients and progressed in two patients for whom surgical intervention was carried out. Six patients developed osteoradionecrosis and were treated with antibiotics for six months; one patient had progression and was sent for surgical salvage.

**Figure 4:**
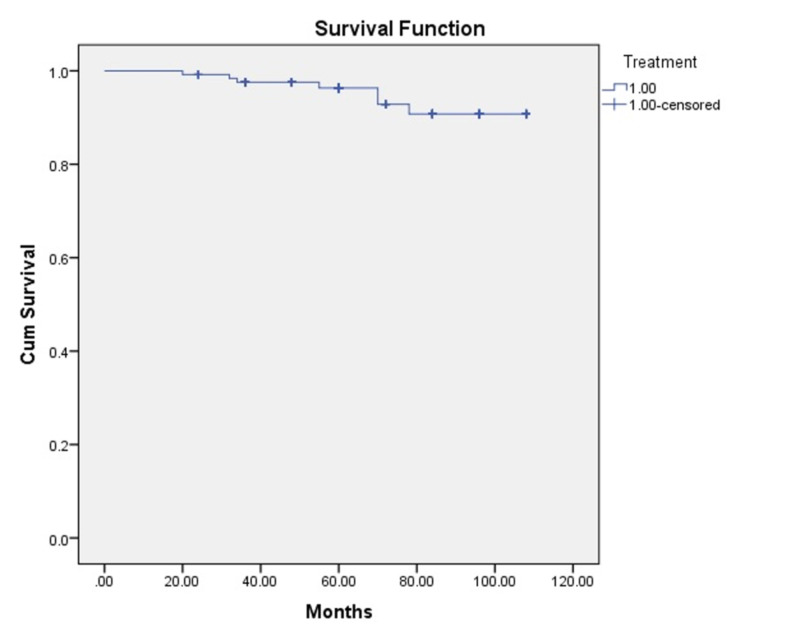
Kaplan-Meier survival analysis with time plotted in months Cum: cumulative

In our study, 17% of patients with stage II and 22.8% with stage III had residual disease. Patients with residual disease were sent for surgical salvage. Seven patients had progressive disease and were not amenable to surgical salvage. They received palliative chemotherapy with cisplatin and paclitaxel. Most of the communications were done via postal means. Despite multiple postal reminders, patients who were referred for surgical salvage did not turn up, and the records of salvage procedures could not be retrieved. Of the 125 patients, 11 patients died: seven due to disease progression and four due to non-cancerous causes. Two patients had a sudden myocardial infarction and died but had achieved good local control. One patient developed sputum-positive pulmonary tuberculosis after three years. He developed massive hemoptysis and died. One patient developed a second tumor from the primary in the brainstem, subsequently developed quadriplegia, and succumbed to the illness. The Kaplan-Meier survival analysis with time plotted in months is presented in Figure 4.

## Conclusions

Brachytherapy catheter implantation and planning can play an important role in ensuring adequate dose coverage to the tumor. Good brachytherapy technique and meticulous planning can minimize toxicity with better tumor control and achieve high local control rates. In treating early oral cavity cancers, brachytherapy with or without EBRT should be considered as an alternative to surgical procedure as it can achieve organ preservation while providing good functional outcomes.
